# Acute shortage of iodinated contrast media: implications and guidance for neurovascular imaging and intervention

**DOI:** 10.1007/s00234-022-02999-6

**Published:** 2022-06-18

**Authors:** Daniel P. O. Kaiser, Mohamad Abdalkader, Anne Berberich, Peter B. Sporns, Thanh N. Nguyen

**Affiliations:** 1 Else Kröner Fresenius Center for Digital Health, TU Dresden, Dresden, Germany; 2 Institut Und Poliklinik Für Diagnostische Und Interventionelle Neuroradiologie, Universitätsklinikum Carl Gustav Carus Dresden, Technische Universität Dresden, Fetscherstraße 74, 01307 Dresden, Germany; 3 Department of Radiology, Boston Medical Center, Boston, MA USA; 4 Department of Neurology, Heidelberg University Hospital, Heidelberg, Germany; 5 Department of Neuroradiology, Clinic of Radiology and Nuclear Medicine, University Hospital Basel, Basel, Switzerland; 6 Department of Diagnostic and Interventional Neuroradiology, University Medical Center Hamburg-Eppendorf, Hamburg, Germany; 7 Department of Neurology, Radiology Boston Medical Center, Boston, MA USA

In April 2022, the long-running lockdown in Shanghai, China, due to the COVID-19 pandemic led to significant restrictions on the production of iodinated contrast media (ICM) of a major supplier [1]. As other suppliers’ stocks were also becoming depleted, this resulted in a global shortage of ICM. In facilities with depleted stocks, rationing of ICM supplies could lead to reduced availability of diagnostic and interventional procedures, which is expected to last until August 2022.

In the realm of acute neurovascular disease, computed tomography angiography (CTA), CT perfusion (CTP), and diagnostic and interventional digital subtraction angiography (DSA) are essential for emergency treatment decision-making, treatment, and follow-up. A shortage of ICM for these examinations may place patients at risk for delayed or deferral of life-saving interventions.

In this commentary, we present strategies to minimize ICM use in daily routine and to conserve resources for essential examinations. These strategies are not exhaustive or prescriptive and should not result in suboptimal image quality nor should they compromise patient care.

**Reduction of examinations** by qualified evaluation of indication and in collaboration with referring physicians may be a strategy to conserve ICM resources or address their shortage.

Potential diagnostic guides are the presence of non-contrast CT (NCCT) hyperdense artery sign in a patient with the appropriate clinical stroke syndrome, which may serve as a surrogate for the presence of large-vessel occlusion (LVO), potentially allowing for bypass of CTA and direct to angiography. Deep learning software or artificial intelligence may also aid in the detection of core infarct, LVO, and bypass CTA and CTP altogether as algorithms are developed [2, 3]. CTP may be redundant in a stepwise diagnostic approach if existing information is reliable to inform treatment. Even in patients with late-presenting large-vessel occlusion, NCCT alone may be used as an alternative to CTP to guide thrombectomy [4].

Other strategies for reduction of imaging exams in the acute stroke patient include direct to angiography approach which may be reasonable in patients presenting from the field with high pre-hospital stroke scales [5] or in transfer patients [6–8]. As stroke centers may have automated protocols to conduct CTA and/or CTP for all stroke patients for the detection of LVO [9], there are subgroups of patients where an algorithmic approach to determining the low likelihood of LVO candidates may render the CTA study unnecessary. Patients who have low NIHSS (i.e., isolated facial droop, isolated dysarthria, and isolated dizziness) have a low likelihood of LVO and can be triaged with magnetic resonance imaging (MRI) and magnetic resonance angiography (MRA) protocols. Patients with completed territorial infarctions (i.e., complete middle cerebral artery infarction) may not need CTA nor CTP imaging as this will likely not change management. These interpretations and reduction of imaging decisions for the acute stroke patient would require a physician to be knowledgeable of the patient’s brief history and exam, interpret the images in the control room while the patient is in the CT scan, to make step-wise triage decisions.

In patients who present with subarachnoid hemorrhage (SAH), it is common practice to perform CTA followed by DSA [10]. Advantages of performing CTA in the workup of SAH include aneurysm treatment planning (i.e., the patient is intubated up front in the angio suite if they are a good candidate for coiling), external ventricular drain trajectory planning, and in high-risk patients, understanding the atherosclerotic burden with catheterization risk. However, in other centers, it is common practice to perform CT followed by direct to four-vessel angiography for aneurysm detection and treatment, which obviates an extra contrast study (i.e., CTA) altogether.

**Alternative imaging modalities** such as MRI or MRA can replace contrast CT examinations in most cases for the head and neck. However, most centers have waiting lists for this modality. The availability of MRI could be optimized by deferring other examinations and implementation of a 24/7 on-call duty. Despite the limited study situation, MRI, if it can be performed without substantial delay, may also be considered for evaluation of patients before thrombectomy in high-performing centers [11]. In patients with unruptured intracranial aneurysms, 3D time of flight MRA (rather than CTA head) would likely suffice in the majority of patients to characterize the aneurysm, facilitate treatment decision-making, and inform long-term aneurysm follow-up [12].

Moreover, Doppler ultrasonography is a valuable alternative for examinations of extracranial vessels, particularly in a patient with a transient ischemic attack (TIA) in whom the likelihood of LVO detection is low. Transcranial Doppler ultrasonography may be useful for monitoring patients with higher-grade SAH and for the development of cerebral vasospasm [13].

**Deferral of imaging** without risk to the patient is possible for most elective examinations, particularly in patients with an unruptured intracranial aneurysm or stable blunt cerebrovascular injury. For example, a follow-up DSA, MRA, or CTA after aneurysm treatment can be safely deferred [14].

**Tailoring ICM dose** for CTA and DSA with aliquots based on a patient’s weight or region of interest may help reduce contrast waste. Reducing ICM dose in conjunction with low kVp or dual-energy protocols to improve contrast conspicuity in CTA and CTP are also important practices in daily routine. As contrast volume may be predictable with imaging protocols and diagnostic or interventional studies, having variable-sized contrast bottles may facilitate tailoring the radiological exam close to the required contrast volume. If only large ICM vials are available, repackaging of the large vials into smaller vials should be discussed with the pharmacy department to help reduce waste.

Not infrequently, for catheter angiographic studies, the power injector is loaded with ICM, and a separate contrast bottle is used for hand injections. Advanced communication with the technologist regarding the preferred route of contrast administration (i.e., power vs hand injection) is important to limit contrast waste.

**The procedural approach** to address contrast shortage comprises a careful review of prior imaging by the neurointerventionalists, as ought to be prepared in routine clinical practice. Knowledge of anatomy, the anatomical variants, the location of the vascular lesion(s) and the side of the pathology (intracranial hemorrhage) from prior imaging can limit ICM usage during the procedure by decreasing unnecessary runs and/or projections. During the procedure, neurointerventionalists should aim to use the minimum number of injections to answer the diagnostic question, but not to leave any potential relevant question unanswered, and lesser contrast volume in each injection similar to the low-contrast protocol used in chronic kidney disease patients [15]. For DSA, optimizing the positioning of the detector, table height, and use of filters to improve image quality are practices that ought to be routine. If not already done, the minimum concentration of ICM diluted with saline should be tested that would permit reliable and optimal visualization of the angiographic images. Biplane angiography can reduce contrast usage by acquiring two images for one injection. Post-acquisition image rendering using ICM, brightness, and edge post-processing tools can optimize image quality to compensate for the decrease in the amount of ICM used. The use of automated power injectors can reduce the amount of ICM used by setting a maximum dose of ICM delivered with any injection. The use of smaller catheters (i.e., 3.2 French or 4 French) can also reduce the ICM volume without compromising the quality of the images, especially when used with a power injector [16].

Neurointerventionalists should refrain from vertebral artery injection if the indication is not related to posterior fossa pathology or just injecting one vertebral artery with goal to reflux into the contralateral vertebral artery to visualize the contralateral posterior inferior cerebellar artery. They should also forgo injecting the external carotid arteries (ECA) if there is no suspicion of ECA pathology or dural arteriovenous fistula. If the angiogram is done for aneurysm treatment planning or for post-treatment follow up, providers should consider MRA rather than CTA or DSA. If DSA is needed, a single vessel angiogram should be adequate in most cases. Three dimensional angiography acquisition with volume rendering reconstruction can be obtained for treatment planning rather than obtaining multiplane angiographic views [17]. For spinal angiography, providers should avoid the contrast “puffs” to locate the radicular arteries and rely more on the tactile feedback when engaging the origins of radicular/segmental arteries.

**Interdisciplinary collaboration** with Pharmacology and other departments (i.e., neurology, neurosurgery, radiology, radiation oncology, nuclear medicine, cardiology, vascular surgery, urology, etc.) is essential to prioritize and optimize ICM usage at the facility.

**Building redundancy** in supply with multiple manufacturers for hospitals may facilitate future preparations and buffer supplies in the event of supply chain shortage of one company, as had been encountered with certain health care resources during the pandemic. Supply chains may be an ongoing problem amidst an ongoing pandemic or post-pandemic era, but other circumstances (e.g., war and natural disasters) can also cause shortage.

## Conclusion

More than two years after the onset of the COVID-19 pandemic, we are confronted with the tangible reality of a heavy reliance on ICM in the care of patients presenting with neurovascular disease. While the shortage of ICM is expected to be temporary, continued conservation and evaluation in the use of ICM is important even after correction of the shortage, to conserve essential clinical resources to patients who need it most. A change in practice should be adapted to the available resources of an institution. Emergent and non-emergent preparedness in the allocation of healthcare resources is important not only amidst an impending crisis [18, 19], but also for averting potential future crises in the immediate and long-term.
